# Geographical Variation in Health Spending Across the US Among Privately Insured Individuals and Enrollees in Medicaid and Medicare

**DOI:** 10.1001/jamanetworkopen.2022.22138

**Published:** 2022-07-20

**Authors:** Zack Cooper, Olivia Stiegman, Chima D. Ndumele, Becky Staiger, Jonathan Skinner

**Affiliations:** 1 Yale School of Public Health, Yale University, New Haven, Connecticut; 2 Department of Economics, Yale University, New Haven, Connecticut; 3 Tobin Center for Economic Policy, Yale University, New Haven, Connecticut; 4 The National Bureau of Economic Research, Cambridge, Massachusetts; 5 Stanford School of Medicine, Stanford University, Stanford, California; 6 The Dartmouth Institute for Health Policy and Clinical Practice, Lebanon, New Hampshire; 7 Department of Economics, Dartmouth College, Hanover, New Hampshire

## Abstract

**Importance:**

Little is known about small-area variations in health care spending and utilization across the 3 major funders of health care in the US: Medicare, Medicaid, and private insurers.

**Objective:**

To measure regional health spending and utilization across Medicare, Medicaid, and the privately insured; to observe whether there are regions that are simultaneously low spending for all 3 payers; and to determine what factors are correlated with regional spending and utilization by payer.

**Design, Setting, and Participants:**

Observational cross-sectional analysis of the US health system in 2016 and 2017 for 241 of 306 hospital referral regions (HRRs) and 2 states. Participants include individuals with employer-sponsored coverage from Aetna, Humana, or UnitedHealth; individuals with Medicaid fee-for-service coverage in 2016 and 2017; and individuals with Medicare coverage. The analysis was carried out from January 2020 to May 2022.

**Main Outcomes and Measures:**

Spending per beneficiary and inpatient days per beneficiary by payer and overall.

**Results:**

The data include 25 381 167 individuals with employer-sponsored coverage, 69 891 299 with Medicaid coverage in 2016 and 2017, and 26 711 426 individuals with Medicare fee-for-service coverage. The percentage of enrollees who identified as female was 54.1% in the Medicaid program, 56.2% in the Medicare program, and 50.4% in private insurance. The mean (SD) age was 26.9 (21.8) years for Medicaid and 75.0 (7.9) years for Medicare enrollees; for private insurance enrollees, just age brackets were reported: 18 to 24 years (15.9%), 25 to 34 years (24.2%), 35 to 44 years (21.3%), 45 to 54 years (20.8%), and 55 to 64 years (17.8%). In 2017, the mean (SD) HRR-level spending per beneficiary was $4441 ($710) for private insurance, $10 281 ($1294) for Medicare, and $6127 ($1428) for Medicaid. Across HRRs, the correlation coefficients and 95% CIs were 0.020 (−0.106 to 0.146; *P* = .76) for private insurance and Medicare spending, 0.213 (0.090 to 0.330; *P* < .001) for private insurance and Medicaid, and 0.162 (0.037 to 0.282; *P* < .01) for Medicare and Medicaid. Just 3 HRRs (Boulder, Colorado; Bloomington, Illinois; and Olympia, Washington) were in the lowest spending quintile for all 3 insurance programs; 4 HRRs were in the highest (The Bronx, New York; Manhattan, New York; White Plains, New York; and Dallas, Texas). By contrast, the correlation coefficients and 95% CIs for utilization, measured in hospital days, were 0.465 (0.361 to 0.559; *P* < .001) for private insurance and Medicare, 0.527 (0.429 to 0.612; *P* < .001) for private insurance and Medicaid, and 0.278 (0.157 to 0.390; *P* < .001) for Medicare and Medicaid.

**Conclusions and Relevance:**

These findings suggest that payer-specific factors are correlated with health spending variation among Medicare beneficiaries, Medicaid beneficiaries, and the commercially insured and that payer-specific policies will be necessary to improve efficiency in the US health sector.

## Key Points

**Questions:**

Is regional health care spending correlated with insurance type (ie, Medicare, Medicaid, and private insurance), are there regions that are low-spending across all 3 payers, and are similar factors correlated with regional spending by payer?

**Findings:**

In this cross-sectional study, there was a low correlation in regional spending across the payers, and only 3 regions were simultaneously in the lowest quintile of spending for each payer. There were no regional correlates of spending that went in the same direction and were significant across all payers.

**Meaning:**

These findings suggest that payer-specific strategies will likely be necessary to raise efficiency in the US health care system.

## Teaser

This cross-sectional study evaluates geographic variation in US health spending among privately insured individuals and enrollees in Medicaid and Medicare.

## Introduction

The US spends approximately $3.8 trillion annually on health care and, by most accounts, is inefficient compared with health systems in other nations.^1^ The US health system is also unusual in that it is composed of multiple, separate funders of health care services, each governed by distinct regulations, market dynamics, and mechanisms for determining the prices set by hospitals and health systems. The 3 dominant payers, Medicare (a federally run program that covers individuals aged 65 years and older and individuals with disabilities), Medicaid (a state-run program funded by states and the federal government that covers individuals under certain income thresholds that vary by state), and private insurers (which provide both individual and employer-sponsored coverage and are subject to state and federal regulations), covered 14.2%, 19.8%, and 49.6% of the population, respectively, in 2019.^2^

In the past, policy makers have singled out a particular region as having differentially efficient health care according to its performance delivering care to a single payer segment (eg, a region’s spending on Medicare beneficiaries).^3^ However, regarding a region as efficient according to the region’s spending on 1 payer segment presupposes that regional spending and the efficiency of care is correlated across payers. Unfortunately, because of data limitations, there has never been a nationwide, small-area analysis of variation in overall spending and utilization that captures data from the 3 major payers of US health care. Although recent work has highlighted the low correlation in regional variation in Medicare spending and private health spending, past issues with data quality have limited the analysis of Medicaid data.^4,5,6,7^

In this study, we use private insurance claims from Aetna, Humana, and UnitedHealthcare through the Healthcare Cost Institute (HCCI) to study the privately insured with employer-sponsored coverage, fee-for-service Medicare claims data to assess the Medicare program, and newly released Medicaid claims data from the Transformed Medicaid Statistical Information System Analytic Files that cover fee-for-service and managed care beneficiaries to analyze Medicaid spending. We use these data to build and analyze a composite measure of regional health spending that incorporates spending from the Medicare program, Medicaid program, and private insurers. We seek to observe whether there are regions that are simultaneously low-spending across all 3 payers, analyze the correlation in spending and inpatient utilization across the 3 payers, and then determine whether the correlates of regional spending are similar across the 3 payers.

We hypothesize that differences in Medicaid reimbursement rates and private insurance prices will lead to considerably more variation in Medicaid and private spending across hospital referral regions (HRRs) than for Medicare, where prices are administered at the federal level. This, in turn, will lead to a low correlation in spending across payers and few regions that are simultaneously low spending across all 3 payer segments. By contrast, for health care utilization, we hypothesize that factors associated with both the demand for health care (eg, health risk factors and income) and supply factors (eg, physician supply and hospital beds) would exhibit similar associations across the 3 insurance programs.

## Methods

### Data

This project has been approved by the Dartmouth and Yale institutional review boards, which also waived the requirement for obtaining informed consent because the claims data was deidentified and not collected for this study. This study followed the Strengthening the Reporting of Observational Studies in Epidemiology (STROBE) reporting guideline. Our primary data come from 3 sources. First, we used data from HCCI, which is composed of private insurance claims for individuals with employer-sponsored plans from Aetna, Humana, and UnitedHealthcare. It covers 29.2% of individuals in the US with employer-sponsored insurance in 2017. The data capture all health care utilization excluding pharmacy claims. See the eMethods in the Supplement for the detailed HCCI sample restrictions.

Second, we used the 100% sample of Medicare fee-for-service claims data from the Dartmouth Atlas for 2017. The Medicare claims capture the universe of health care services, excluding pharmacy claims, delivered to the 38 667 830 individuals enrolled in Parts A and B of the Medicare fee-for-service program in 2017. This represents approximately 66.15% of Medicare beneficiaries nationwide, since 33.85% of Medicare beneficiaries are enrolled in a privately administered Medicare Advantage plan.^8^

Third, we used the Transformed Medicaid Statistical Information System Analytic Files data, managed by the Centers for Medicare and Medicaid Services (CMS). Medicaid is a state-run program, so data quality varies by state. The online Data Quality (DQ) Atlas provides guidance on the data quality for a range of measures, and ranks states’ data as low concern, medium concern, high concern, and unusable.^9^ We limited our analysis to states that are rated by the DQ Atlas as either low concern or medium concern. To maximize the number of HRRs available for analysis, we relied primarily on 2017 Medicaid data, but use 2016 data (inflation-adjusted into 2017 dollars) for 3 states when the state-level quality was of high concern or unusable in 2017 and was of low concern or medium concern in 2016. Collectively, the 41 states rated by the DQ Atlas as having data of low or medium concern in either 2016 or 2017 form the analytical sample. The analysis of Medicaid data was also limited to individuals with full benefits (eg, we excluded individuals who exclusively receive maternity benefits or who have restricted coverage).

### Constructing Key Variables

We constructed measures of spending and inpatient days per beneficiary by payer (detailed methods for constructing spending measures are included in the eMethods in the Supplement). Our unit of analysis was the HRR; the exception is where we replace the HRR boundaries with state boundaries for 2 states (Vermont and Wyoming) because they lacked Medicaid zip code information.^10^

The composite measures of spending per beneficiary and inpatient days per beneficiary per HRR were constructed using a weighted mean of private insurance data, Medicare data, and Medicaid data. Observations were weighted by each state’s 2017 share of employer-sponsored, Medicare, and Medicaid beneficiaries.^2^ For HRRs that cross multiple states, we calculated the mean for these states’ shares, weighted by the share of each state’s population in the HRR. Spending measures were risk-adjusted by insurance type and by age and sex of enrollees.

### Statistical Analysis

We correlated spending per beneficiary and inpatient days per beneficiary with a variety of regional measures of demand, supply, local market characteristics, and prices set by hospitals and health systems. A large health economics literature has described key demand-side and supply-side drivers of spending and utilization, which we integrated as the main correlates in our analysis.^4,5,11,12,13^ The eMethods in the Supplement includes a full list of the correlates. For the bivariate correlation analysis, all variables were standardized to have a mean of 0 and SD of 1, then analyzed using ordinary least squares.

We considered several sensitivity analyses. First, we included all Medicaid beneficiaries, including those without full coverage. Second, rather than weight our composite spending measure by state-level coverage by payer, we weighted the overall spending measure by the national share of each of the 3 main payers. Third, we showed our spending and quantity correlations between Medicare and the privately insured are robust even when we included areas excluded because of poor Medicaid data quality. Fourth, rather than manually selecting the bivariate correlates of health spending and utilization, we included characteristics selected by a least absolute shrinkage and selection operator (LASSO) regression on composite spending (see eFigure 3 in the Supplement). Finally, we present bivariate correlates using a Bonferroni correction rather than false discovery rate q-values. Statistical significance was defined as a 2-sided *P* < .05. All analysis was carried out using Stata/MP statistical software version 17 (StataCorp). Data were analyzed from January 2020 to May 2022.

## Results

The data used in this analysis include 25 381 167 individuals with employer-sponsored coverage in 2017, 69 891 299 with Medicaid coverage in 2016 and 2017, and 26 711 426 individuals with Medicare fee-for-service coverage. The percentage of enrollees who identified as female was 54.1% in the Medicaid program, 56.2% in the Medicare program, and 50.4% in private insurance. The mean (SD) age was 26.9 (21.8) years for Medicaid and 75.0 (7.9) years for Medicare enrollees; for private insurance enrollees, just age brackets were reported: 18 to 24 years (15.9%), 25 to 34 years (24.2%), 35 to 44 years (21.3%), 45 to 54 years (20.8%), and ages 55 to 64 years (17.8%). Our sample includes analysis of 241 of 306 HRRs and 2 states. (The results are qualitatively unchanged when we carry out the analysis on the full sample of HRRs available via data from HCCI and the Medicare program.) Collectively, our analysis is based on $683 billion in total health spending across Medicare, Medicaid, and the privately insured.

Health care spending per beneficiary varied significantly within and across payers (Table 1). In 2017, mean (SD) HRR-level private insurance spending per beneficiary was $4441 ($710), Medicare mean (SD) spending per beneficiary was $10 281 ($1294), and Medicaid mean (SD) spending per beneficiary was $6127 ($1428). The weighted overall mean (SD), which accounts for the number of beneficiaries in each program, was $5782 ($622) per beneficiary.

**Table 1.** HRR-Level Spending and Inpatient Days per Beneficiary Across Payers

Payer	HRRs	Mean (SD)	Coefficient of variation	Minimum	10th Percentile	25th Percentile	50th Percentile	75th Percentile	90th Percentile	Maximum
Age- and sex-adjusted spending per beneficiary^a^										
Private	243	4441 (710)	0.160	2655	3616	3933	4369	4924	5469	6742
Medicare	243	10 281 (1294)	0.126	7654	8842	9262	10 093	11 078	12 073	15 186
Medicaid	243	6127 (1428)	0.233	2692	4440	5031	6018	7187	8079	10 472
Composite^b^	243	5782 (622)	0.108	4184	5018	5268	5766	6242	6602	7705
Age- and sex-adjusted inpatient days per beneficiary^c^										
Private	243	0.21 (0.04)	0.180	0.11	0.16	0.19	0.21	0.23	0.25	0.32
Medicare	243	1.30 (0.27)	0.207	0.69	0.98	1.10	1.30	1.49	1.66	2.25
Medicaid	243	0.56 (0.17)	0.301	0.20	0.33	0.42	0.56	0.68	0.76	1.01
Composite^b^	243	0.47 (0.09)	0.195	0.28	0.34	0.40	0.47	0.54	0.60	0.70

Abbreviation: HRR, hospital referral region.

^a^ Spending per beneficiary is age-adjusted and sex-adjusted by payer using indirect standardization and is presented in 2017 US dollars at the HRR level.

^b^ Composite spending and composite inpatient days are means of private, Medicare, and Medicaid spending and inpatient days, respectively, weighted by the state-level share of the population that is insured by the payer.

^c^ Inpatient days per beneficiary are age-adjusted and sex-adjusted using indirect standardization and are presented at the HRR level.

The Medicaid program exhibited the most variation in spending per beneficiary across HRRs, with a coefficient of variation of 0.233. The coefficient of variation for private insurance was 0.160. For Medicare, the coefficient of variation was 0.126. Finally, the coefficient of variation for overall or composite spending per beneficiary was 0.108.

The mean (SD) number of inpatient days was 1.30 (0.27) for Medicare beneficiaries, 0.21 (0.04) for the privately insured, and 0.56 (0.17) for Medicaid beneficiaries (Table 1). Even after adjusting for age and sex, there was substantial variation in inpatient days per beneficiary within payers across HRRs. The coefficient of variation in inpatient days per beneficiary was 0.180 for the privately insured, 0.207 for Medicare beneficiaries, and 0.301 for Medicaid beneficiaries. For composite hospital utilization, the coefficient of variation was 0.195.

The correlation coefficient and 95% CI between HRR level spending was 0.020 (−0.106 to 0.146; *P* = .76) for private insurance and Medicare, 0.213 (0.090 to 0.330; *P* < .001) for private insurance and Medicaid, and 0.162 (0.037 to 0.282; *P* < .011) for Medicare and Medicaid (Table 2; eFigure 1 in the Supplement for scatter plots of these correlations). By contrast, there was a higher correlation across payers in inpatient days per beneficiary. As shown in Table 2, the correlation coefficient and 95% CI was 0.465 (0.361 to 0.559; *P* < .001) for private insurance and Medicare, 0.527 (0.429 to 0.612; *P* < .001) for private insurance and Medicaid, and 0.278 (0.157 to 0.390; *P* < .001) for Medicare and Medicaid.

**Table 2.** Correlation of Spending per Beneficiary and Inpatient Days per Beneficiary Across Payers

	Private	Medicare
Correlation of spending per beneficiary^a^		
Private	1.000	
Medicare	0.020	
Medicaid	0.213^b^	0.162^c^
Correlation of inpatient days per beneficiary^d^		
Private	1.000	
Medicare	0.465^b^	
Medicaid	0.527^b^	0.278^b^

Abbreviation: HRR, hospital referral region.

^a^ HRR-level spending measures are age-adjusted and sex-adjusted using indirect standardization, and inflation-adjusted to 2017 US dollars.

^b^ *P* < .01.

^c^ *P* < .05.

^d^ HRR-level inpatient days per beneficiary are age-adjusted and sex-adjusted using indirect standardization.

In eTable 1 in the Supplement, we show the correlation between overall health care spending per beneficiary by payer and inpatient days per beneficiary by payer. In the Medicare program, where reimbursements to hospitals and health systems are regulated, there was a correlation and 95% CI of 0.665 (0.589-0.730; *P* < .001) between HRR-level Medicare spending per beneficiary and Medicare inpatient days per beneficiary. By contrast, among the privately insured, where reimbursements to hospitals and health systems are market determined, there was only a 0.131 (0.005-0.253; *P* < .041) correlation between overall private spending per beneficiary and inpatient days per privately insured beneficiaries across HRRs. In the Medicaid sample, where prices set by hospitals and health systems are regulated in some areas and negotiated in others, the correlation and 95% CI between HRR-level Medicaid spending per beneficiary and Medicaid inpatient days per beneficiary was 0.347 (0.231-0.453; *P* < .001).

**Figure 1.**
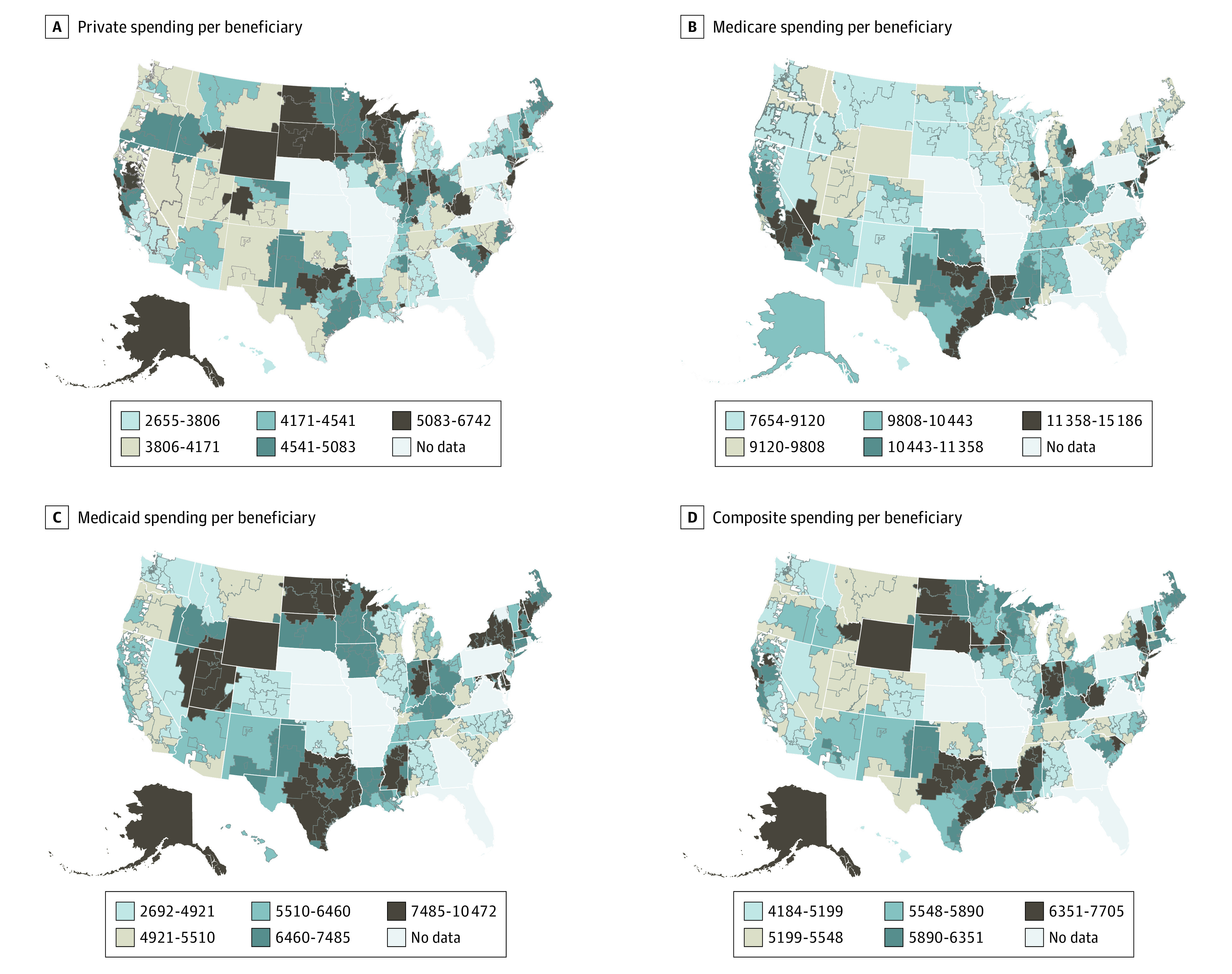
Spending per Beneficiary by Payer All spending measures are age- and sex-adjusted using indirect standardization and presented in 2017 US dollars. Composite spending and composite inpatient days are means of private, Medicare, and Medicaid spending and inpatient days, respectively, weighted by the state-level share of the population that is covered by the payer.

Figure 1 maps spending per beneficiary among the privately insured, Medicare beneficiaries, and Medicaid beneficiaries. Because of the low correlations in spending per beneficiaries across payers, as shown in Table 2, there were few regions that are universally high spending or low spending, with just 3 HRRs (Boulder, Colorado; Bloomington, Illinois; and Olympia, Washington) in the lowest quintile of spending per beneficiary across all 3 insurance programs, and 4 HRRs in the highest (The Bronx, New York; Manhattan, New York; White Plains, New York; and Dallas, Texas). In eTable 2 in the Supplement, we present the 20 highest- and lowest-spending HRRs on a composite basis and by payer.

**Figure 2.**
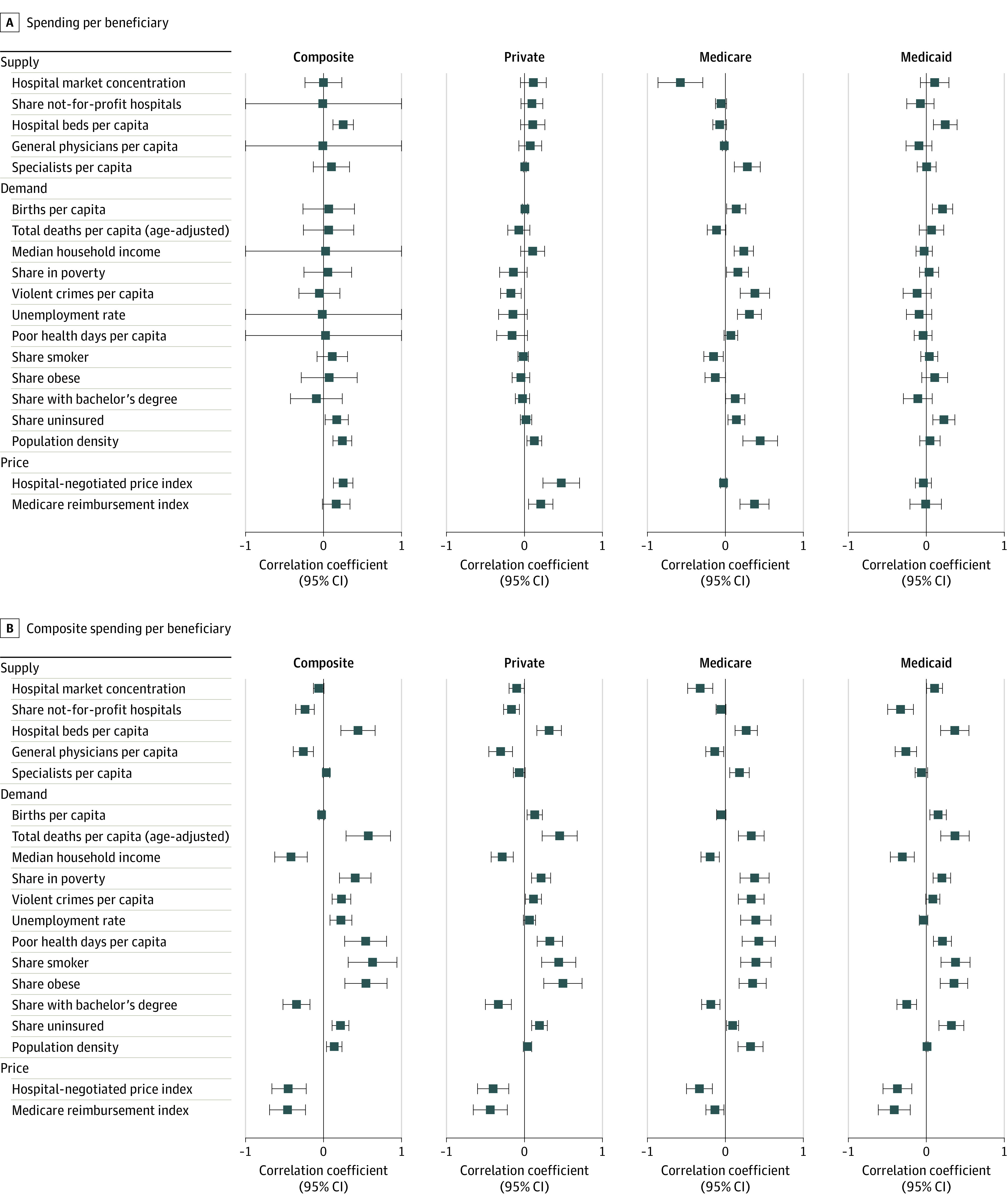
Bivariate Correlates of Spending and Inpatient Days per Beneficiary Across Payers Using Sharpened False Discovery Rate (FDR) *q* Values The bivariate correlates include 95% CIs under sharpened FDR *q* values. Hospital referral region–level spending and inpatient days per beneficiary are age-adjusted and sex-adjusted using indirect standardization. Composite spending and days are a mean of private, Medicare, and Medicaid spending and days, respectively, weighted by the state-level share of the population that is insured by the payer. Note that 0.0001 is the minimum for sharpened FDR *q* values.

Figure 2 presents bivariate correlates of health spending per beneficiary and inpatient days per beneficiary by payer and for the composite sample with a range of supply-side, demand-side, and hospital price measures. SEs have been adjusted using sharpened false discovery rate *q* values.^13^ For spending (Figure 2A), the largest bivariate correlates of composite spending were hospital negotiated prices (correlation coefficient, 0.25; *q* < 0.01), hospital beds per capita (correlation coefficient, 0.25; *q* < 0.01), population density (correlation coefficient, 0.24; *q* < 0.01), the share of the population that is uninsured (correlation coefficient, 0.17; *q* < 0.05), and the Medicare reimbursement level (correlation coefficient, 0.16; *q* < 0.10). Most other measures have correlations that were more modest in magnitude and not significantly different from 0. There was no significant bivariate correlation between overall composite health spending and deaths per capita. When these correlations were performed separately by payer, there was no characteristic that was universally positively or negatively significantly correlated with overall spending per beneficiary. This remained true even when we did not adjust our SEs for multiple hypothesis testing (eFigure 2 in the Supplement).

By contrast, a range of demand-side and supply-side characteristics exhibited bivariate associations with inpatient days per beneficiary among each payer. Factors that might indicate higher demand for health care—the share of patients who smoke (correlation coefficient, 0.63; *q* < 0.01), the share with obesity (correlation coefficient, 0.54; *q* < 0.01), and poor health days per capita (correlation coefficient, 0.54; *q* < 0.01)—were positively associated with hospital days. Inpatient days per capita were negatively associated with median household income (correlation coefficient, −0.42; *q* < 0.01). Unlike spending, hospital days were associated with mortality rates (correlation coefficient, 0.57; *q* < 0.01). Hospital beds per capita were positively correlated with inpatient days per beneficiary (correlation coefficient, 0.44; *q* < 0.01). When these correlates are considered separately by payer, there was considerable consistency across the correlates. Results are presented in eFigure 4 in the Supplement; none of these sensitivity analyses affected our results.

## Discussion

In this cross-sectional study, we present a national analysis of US regional variation in health spending and utilization for enrollees in the Medicare program, the Medicaid program, and private insurance plans offering employer-sponsored coverage. To our knowledge, this work is the first to analyze the correlation in regional spending between Medicaid and Medicare and Medicaid and the privately insured, and the first to identify whether there are regions that are consistently low-spending or high-spending across all 3 main funders of US health services.

This research has 4 main findings. First, there was substantial variation in spending across all 3 payers. Ultimately, the Medicaid and privately insured populations exhibited more variation in spending across regions than was present in the Medicare program. This likely reflects the fact that, whereas the Medicare program relies on regulated payments to hospitals and health systems, prices for private and Medicaid managed care (70% of Medicaid) are generally market-determined and vary substantially across regions.^4,5,14,15^

Second, there was a low correlation in spending within regions across the Medicare program, the Medicaid program, and the privately insured. These results are consistent with an earlier set of studies^4,7^ that also documented that the correlation between Medicare and private insurance expenditures was low. The low correlation (0.162) in within-region spending between the Medicare and Medicaid programs is striking, since both are largely funded by the federal government through the Centers for Medicare and Medicaid Services. However, although the Medicaid program is largely federally funded, it is administered by states, which take distinct approaches to organizing and regulating the program. A byproduct of this low correlation is that there are only 3 HRRs nationwide that are in the bottom quartile of spending for each major payer.

Third, despite the lack of correlation in health care spending, regional variation in utilization, measured by inpatient bed days per beneficiary, was correlated across Medicare, Medicaid, and the privately insured. Indeed, the correlates of inpatient utilization—indicators of population health (mortality, smoking, and obesity), hospital beds per capita, and rates of reimbursement to hospitals and health systems—were similar in direction and magnitude across the 3 payers. This finding is consistent with previous research showing that for utilization of health care services (rather than spending per se), there is a distinct signature of regional health care practice.^11,16,17^

Fourth, there were no correlates of variation in spending that were consistently signed and significant across the 3 payers. This highlights that payer-specific factors likely lead to variation in health spending among Medicare beneficiaries, Medicaid beneficiaries, and the privately insured. For the privately insured, regions with high prices tend to have higher spending. For the Medicare program, regions with higher spending have more specialist physicians per capita. For the Medicaid program, regions with higher spending have more hospital beds per capita and more births per capita.

These findings have substantial implications for health policy. First, they suggest that analysts cannot understand the overall performance of a region by examining that region’s performance for 1 payer. Second, the presence of payer-specific determinants of spending variation suggests that policy makers should consider focusing on payer-specific policies. This could include, for example, addressing unwarranted quantity variation in the Medicare population or introducing policies in private markets that lead to more efficient hospital pricing. Third, the wide variations in health spending across payers within regions also suggests that policy makers should be cautious about the unintended local spillover effects of their policies. For example, policies that target variation in quantities in the Medicare population by integration of hospitals and health systems could have the unintended effect of raising provider prices and spending among the privately insured.

### Limitations

There are several limitations to our work. First, because of Medicaid data quality, we are missing spending and utilization data for 65 HRRs, which comprise 22% of the population. To the extent that the poor data quality is random, this need not affect our broad conclusions. However, if states missing from the sample are nonrandom, we may either underestimate or overestimate variation. Second, the HCCI data covers only a subset of the privately insured population. Thus, we must assume that average price levels in specific markets for other private insurance carriers are correlated with the large insurance carriers in the HCCI sample. Third, to analyze Medicare spending, we rely on Medicare fee-for-service claims. In 2017, approximately a third of Medicare beneficiaries were enrolled in a Medicare Advantage plan administered by a private insurer. However, the Medicare Advantage fee schedule is linked to the fee-for-service fee schedule, and prior work has illustrated a high correlation in spending across the 2 populations.^18^ Fourth, we only risk-adjust our spending measures for age and sex of beneficiaries by payer. However, we show how other aspects of demand for care, such as local obesity levels, correlate with our overall and payer-specific spending measures.

## Conclusions

This cross-sectional study documented substantial variation in health care spending and utilization for Medicare, Medicaid, and private insurance plans across small-area regions of the US. Our findings suggest that payer-specific factors are associated with regional variations in spending for Medicare beneficiaries, Medicaid beneficiaries, and the privately insured, suggesting an important role for payer-specific policies to address health spending. Our work highlights the need for future research to better identify the determinants of regional inefficiencies in the US health sector.

## Notes

Supplement.
eMethods

eReferences
**eTable 1.** Correlation of Spending Per Beneficiary and Inpatient Bed-Days Per Beneficiary by Payer **eTable 2.** Highest- and Lowest-Spending HRRs by Payer **eTable 3.** Correlation of Inclusive and Exclusive Medicaid Spending Per Beneficiary and Spending Per Beneficiary Across Payers with an Inclusive Estimate of Medicaid Spending **Figure 1.** Scatter Plots of Correlations of HRR-Level Spending Per Beneficiary Across Payers **eFigure 2.** Bivariate Correlates of Spending and Inpatient Bed-Days Per Beneficiary Across Payers **eFigure 3.** Bivariate Correlates of Spending and Inpatient Bed-Days Per Beneficiary Across Payers with Characteristics Selected by a LASSO Regression **eFigure 4.** Bivariate Correlates of Spending and Inpatient Bed-Days Per Beneficiary Across Payers Using a Bonferroni Correction
