# Error in Figures

**DOI:** 10.1001/jamanetworkopen.2022.30459

**Published:** 2022-08-17

In the Original Investigation titled “Geographical Variation in Health Spending Across the US Among Privately Insured Individuals and Enrollees in Medicaid and Medicare,”^1^ published July 20, 2022, there was an error in the figures. Figure 2B should have been labeled “Inpatient days per beneficiary,” not “Composite spending per beneficiary.” This article has been corrected.^1^
