# Supplementary material for: Geographical Variation in Health Spending Across the US Among Privately Insured Individuals and Enrollees in Medicaid and Medicare
Source: JAMA Netw Open. 2022 Jul 20;5(7):e2222138. doi: 10.1001/jamanetworkopen.2022.22138 (PMC9301520; doi:10.1001/jamanetworkopen.2022.22138)
Supplement: Supplement. — eMethods eReferences eTable 1. Correlation of Spending Per Beneficiary and Inpatient Bed-Days Per Beneficiary by Payer eTable 2. Highest- and Lowest-Spending HRRs by Payer eTable 3. Correlation of Inclusive and Exclusive Medicaid Spending Per Beneficiary and Spending Per Beneficiary Across Payers with an Inclusive Estimate of Medicaid Spending Figure 1. Scatter Plots of Correlations of HRR-Level Spending Per Beneficiary Across Payers eFigure 2. Bivariate Correlates of Spending and Inpatient Bed-Days Per Beneficiary Across Payers eFigure 3. Bivariate Correlates of Spending and Inpatient Bed-Days Per Beneficiary Across Payers with Characteristics Selected by a LASSO Regression eFigure 4. Bivariate Correlates of Spending and Inpatient Bed-Days Per Beneficiary Across Payers Using a Bonferroni Correction [file jamanetwopen-e2222138-s001.pdf]

Supplemental Online Content

Cooper Z, Stiegman O, Ndumele CD, Staiger B, Skinner J. Geographical variation in health spending across the US among privately insured individuals and enrollees in Medicaid and Medicare. *JAMA Netw Open.* 2022;5(7):e2222138.
doi:10.1001/jamanetworkopen.2022.22138

eMethods
eReferences
eTable 1. Correlation of Spending Per Beneficiary and Inpatient Bed-Days Per Beneficiary by Payor
eTable 2. Highest- and Lowest-Spending HRRs by Payor
eTable 3. Correlation of Inclusive and Exclusive Medicaid Spending Per Beneficiary and Spending Per Beneficiary Across Payors with an Inclusive Estimate of Medicaid Spending
Figure 1. Scatter Plots of Correlations of HRR-Level Spending Per Beneficiary Across Payors
eFigure 2. Bivariate Correlates of Spending and Inpatient Bed-Days Per Beneficiary Across Payors
eFigure 3. Bivariate Correlates of Spending and Inpatient Bed-Days Per Beneficiary Across Payors with Characteristics Selected by a LASSO Regression
eFigure 4. Bivariate Correlates of Spending and Inpatient Bed-Days Per Beneficiary Across Payors Using a Bonferroni Correction

This supplemental material has been provided by the authors to give readers additional information about their work.

© 2022 Cooper Z et al. *JAMA Network Open.*

eMethods

HCCI Data and Sample Restrictions

The analysis of Health Care Cost Institute (HCCI) data is limited to individuals aged 18 through 64. We also exclude individuals for whom there is no home zip code, age, or sex information. This eliminates 7.1% of the covered lives in our privately insured sample.

Medicaid Data and Sample Restrictions

The Centers for Medicare and Medicaid Services (CMS) provides an online Data Quality (DQ) Atlas, giving guidance on the data quality for a range of measures in the Transformed Medicaid Statistical Information System (T-MSIS) Analytic Files (TAF), and ranks states' data as "low concern," "medium concern," "high concern" and "unusable". We limit our analysis to state data that are rated by the DQ Atlas as either "low concern" or "medium concern." When states shift from "medium concern" to "low concern" or conversely, we did not detect any discernable difference in reported spending. Our results are also insensitive to whether we include four states marked as low concern overall but higher concern for inpatient stays (Vermont, Maryland, Maine, and North Carolina).

The analysis of Medicaid data is also limited to individuals with full benefits (e.g., we exclude individuals who exclusively receive maternity benefits or who have restricted coverage). This excludes 7,621,967 of 81,515,928 individuals from the analysis. Individuals without reported age or sex are excluded, and individuals with a missing and unimputable eligibility group are also excluded. This results in the exclusion of an additional 0.13% of covered lives. Finally, we exclude individuals missing zip code information, except in the states of Vermont and Wyoming. Zip code information is lacking for a large portion of individuals in these two states, so we analyze these states in their entirety. 30% of Medicaid beneficiaries are not enrolled for a full calendar year, so we weight each member by the fraction of the year they are enrolled.

Construction of Spending Per Beneficiary and Inpatient Days Per Beneficiary

There are modest differences in how we measure spending per beneficiary across each payor. In our privately insured sample, we sum the allowed amounts, which capture the total of the insurer payments and co-pays, across all medical claims annually by member. For the Medicare sample, we also sum across the spending on each claim. In practice, the Dartmouth Atlas measures are limited to what the federal government pays, and thus do not capture copays and deductibles. However, because Part B deductibles are a constant fraction of payments (e.g., a 20 percent coinsurance payment), relative variations across regions are likely to be largely unaffected. Finally, in the Medicaid program, a large portion of Medicaid enrollees are in managed care plans. As a result, our Medicaid spending measure is the sum of both the allowed amounts on medical claims for individuals in Medicaid fee-for-service and the capitated payments that the state Medicaid agency pays for individuals on managed care plans. Because Medicaid imposes virtually no (or very small) copayments, there is no distinction between government payments and allowed amounts.

We sum spending by member and payor annually and weight our hospital referral region (HRR)-level spending by the number of months each individual was enrolled. We then adjust our

© 2022 Cooper Z et al. *JAMA Network Open.*

spending measure, by payor, for age (for private insurance 18-24 and 10-year intervals from age 25-64, for Medicare 5-year intervals from age 65-84 and 85+, and for Medicaid 5-year intervals from age 0-89 and 90+) and sex using indirect standardization.

To measure inpatient days per beneficiary by payor, we rely on the admission date and discharge dates recorded in the inpatient claims. We then sum inpatient days by payor and construct measures of inpatient days per beneficiary per payor, per HRR. We adjust inpatient days per beneficiary by age and sex using the same indirect standardization as above.

Construction of Hospital Concentration Measure and Hospital Negotiated Price Index

To calculate hospital market concentration, each hospital's market is defined as the area within a 30-minute travel time from the hospital, which is measured using data from HERE, a mapping software application. A Herfindahl-Hirschman Index (HHI), which measures market concentration, is calculated for each hospital's market using the sum of hospital beds in the market as the size of the market and a hospital's count of beds as its measure of market share. We average the hospital-level HHIs to the HHR-level. HHIs range from 0 to 10,000 with a HHI of zero representing a 'perfectly competitive' market of many small firms and a HHI of 10,000 representing a monopoly market. To calculate the hospital negotiated price index, private insurance claims for all inpatient care provided to covered 18-to-64-year-olds in American Hospital Association (AHA)-registered facilities from 2017 are retained from the HCCI database. Data are then limited to general medical/surgical hospitals with at least 50 cases. Hospital payments are regressed on hospital fixed effects, a vector of patient characteristics, and a vector of patient diagnoses (DRG) fixed effects. The vector of hospital fixed effects is recovered, and the hospital price index is calculated using the sample means of the patient characteristics and the DRG indicators. Further details on the price index can be found in a previous study.[1]

Linking Data

A county-to-HRR crosswalk maintained by the United States (US) Census (MABLE – 14) is used to convert county-level data (where necessary) to HRR-level data.[2] For counties not available in this crosswalk, a county-to-zip code crosswalk maintained by the US Department of Housing and Urban Development and a zip-to-HRR crosswalk maintained by the Dartmouth Atlas are used.[3,4]

Selection of Correlates

A list of the chosen correlates, along with their sources, is given below.

Births per capita	Area Health Resource Files
General physicians per capita	Area Health Resource Files
Hospital beds per capita	American Hospital Association
Hospital market concentration	American Hospital Association
Hospital negotiated price index	HCCI Claims
Median household income	American Community Survey 5-Year Data
Medicare reimbursement index	Medicare Impact Files

© 2022 Cooper Z et al. *JAMA Network Open.*

Poor health days per capita	County Health Rankings
Population density	American Community Survey 5-Year Data
Share in poverty	American Community Survey 5-Year Data
Share not-for-profit hospitals	American Hospital Association
Share obese	County Health Rankings
Share smoker	County Health Rankings
Share uninsured	Area Health Resource Files
Share with bachelor's degree	American Community Survey 5-Year Data
Specialists per capita	Area Health Resource Files
Total deaths per capita (age-adjusted)	Centers for Disease Control and Prevention (CDC) Wonder
Unemployment rate	American Community Survey 5-Year Data
Violent crimes per capita	County Health Rankings

The variables included in the least absolute shrinkage and selection operator (LASSO) regression are those in the list above plus those in the list below.

Alcohol-impaired driving deaths per capita	County Health Rankings
Ambulatory surgical centers per capita	Area Health Resource Files
Bassinets per capita	Area Health Resource Files
General physicians per capita	Area Health Resource Files
Gini coefficient	American Community Survey 5-Year Data
Health centers per capita	Area Health Resource Files
Hospital deaths per capita	CDC Wonder
Injury deaths per capita	County Health Rankings
Median age	American Community Survey 5-Year Data
Medicaid expansion status	Kaiser Family Foundation
NHSC active sites per capita	Area Health Resource Files
Non-hospital deaths per capita	CDC Wonder
Nursing facilities per capita	Area Health Resource Files
Operating rooms per capita	Area Health Resource Files
Physical therapists per capita	Area Health Resource Files
Premature deaths per capita	CDC Wonder
Public health workers per capita	Area Health Resource Files
Psychiatrists and psychologists per capita	Area Health Resource Files
Rural health clinics per capita	Area Health Resource Files
Sexually transmitted infections per capita	County Health Rankings
Share 65+	American Community Survey 5-Year Data
Share Asian	American Community Survey 5-Year Data
Share Black	American Community Survey 5-Year Data
Share disabled	American Community Survey 5-Year Data
Share drive alone commute	American Community Survey 5-Year Data
Share excessive drinkers	County Health Rankings
Share female	American Community Survey 5-Year Data

© 2022 Cooper Z et al. *JAMA Network Open.*

Share foreign-born	American Community Survey 5-Year Data
Share Hispanic	American Community Survey 5-Year Data
Share in poor/fair health	County Health Rankings
Share on SNAP	Area Health Resource Files
Share physically inactive	County Health Rankings
Share public transport commute	American Community Survey 5-Year Data
Share religious hospitals	American Hospital Association
Share single parent household	American Community Survey 5-Year Data
Share urban	American Community Survey 5-Year Data
Share veteran	American Community Survey 5-Year Data
Share White	American Community Survey 5-Year Data
Share with exercise opportunities	County Health Rankings
Share with a Health Insurance Marketplace plan	Area Health Resource Files
Share without a high school degree	American Community Survey 5-Year Data
Skilled nursing facilities per capita	Area Health Resource Files
Specialists per capita	Area Health Resource Files
Teen birth rate	County Health Rankings
Total deaths per capita	Area Health Resource Files
Years of potential life lost per capita	County Health Rankings

Sensitivity Analyses

We conducted robustness checks of our spending and quantity correlations between Medicare and the privately insured in areas where the Medicaid data are low quality and across the country including those areas. In areas where the Medicaid data are low quality, the correlation between HRR-level Medicare and private insurance spending is 0.052 ($p = 0.69$), and the correlation between HRR-level Medicare and private insurance inpatient bed-days is 0.476 ($p < 0.01$). Across the country, including areas where the Medicaid data are low quality, the correlation between HRR-level Medicare and private insurance spending is 0.034 ($p = 0.55$), and the correlation between HRR-level Medicare and private insurance inpatient bed days is 0.476 ($p < 0.01$).

The results of several additional sensitivity analyses are presented in the eTables and eFigures.

© 2022 Cooper Z et al. *JAMA Network Open.*

eReferences

1. Cooper Z, Craig SV, Gaynor, M, Van Reenen J. The price ain't right? Hospital prices and heath spending on the privately insured. Q J Econ. 2019;134(1): 51-107.
2. Missouri Census Data Center. Geocorr 2014: Geographic correspondence engine. https://mcdc.missouri.edu/applications/geocorr2014.html. Accessed August 2021.
3. Housing and Urban Development's Office of Policy Development and Research. HUD USPS ZIP code crosswalk files. https://www.huduser.gov/portal/datasets/usps_crosswalk.html. Accessed August 2021.
4. Dartmouth Atlas. Supplemental data. https://data.dartmouthatlas.org/supplemental/. Accessed July 2021.

© 2022 Cooper Z et al. *JAMA Network Open.*

eTable 1. Correlation of Spending Per Beneficiary and Inpatient Bed-Days Per Beneficiary by Payor

		Spending[a]		
		Private	Medicare	Medicaid
Inpatient Bed-Days[b]	Private	0.131**		
	Medicare	-0.096	0.665***	
	Medicaid	0.013	0.017	0.347***

[a]HRR-level spending measures are age- and sex-adjusted using indirect standardization, and inflation-adjusted to 2017 USD.
[b]HRR-level inpatient bed-days per beneficiary are age- and sex-adjusted using indirect standardization.

© 2022 Cooper Z et al. *JAMA Network Open.*

eTable 2. Highest- and Lowest-Spending HRRs by Payor[a]

(A) Highest-Spending HRRs by Payor

Private		
1.	Huntington, WV	$6,742
2.	Anchorage, AK	$6,337
3.	Sioux Falls, SD	$5,998
4.	Marshfield, WI	$5,934
5.	Owensboro, KY	$5,918
6.	Minot, ND	$5,909
7.	Rochester, MN	$5,906
8.	San Mateo County, CA	$5,903
9.	Charleston, WV	$5,818
10.	La Crosse, WI	$5,770
11.	Florence, SC	$5,715
12.	Bismarck, ND	$5,680
13.	White Plains, NY	$5,642
14.	Contra Costa County, CA	$5,627
15.	The Bronx, NY	$5,592
16.	Wausau, WI	$5,591
17.	Santa Rosa, CA	$5,590
18.	Manhattan, NY	$5,561
19.	Rapid City, SD	$5,560
20.	Urbana, IL	$5,519

Medicare		
1.	The Bronx, NY	$15,186
2.	Los Angeles, CA	$14,473
3.	Manhattan, NY	$13,810
4.	East Long Island, NY	$13,461
5.	Munster, IN	$13,027
6.	Chicago, IL	$12,915
7.	Detroit, MI	$12,861
8.	San Bernardino, CA	$12,856
9.	Baltimore, MD	$12,846
10.	Flint, MI	$12,823
11.	Newark, NJ	$12,807
12.	Dearborn, MI	$12,608
13.	Wichita Falls, TX	$12,591
14.	Paterson, NJ	$12,382
15.	Camden, NJ	$12,371
16.	Alameda County, CA	$12,352
17.	Blue Island, IL	$12,254
18.	Hackensack, NJ	$12,230
19.	Las Vegas, NV	$12,228
20.	Houston, TX	$12,215

Medicaid		
1.	San Angelo, TX	$10,472
2.	Abilene, TX	$9,825
3.	Bismarck, ND	$9,809
4.	Wilmington, DE	$9,416
5.	Corpus Christi, TX	$9,037
6.	Salisbury, MD	$8,788
7.	Provo, UT	$8,729
8.	Waco, TX	$8,687
9.	Baltimore, MD	$8,584
10.	White Plains, NY	$8,573
11.	Jackson, MS	$8,515
12.	Fargo/Moorhead MN, ND	$8,512
13.	Victoria, TX	$8,485
14.	Oxford, MS	$8,485
15.	Grand Forks, ND	$8,469
16.	Albany, NY	$8,414
17.	Longview, TX	$8,279
18.	Hattiesburg, MS	$8,270
19.	Rochester, NY	$8,239
20.	Gulfport, MS	$8,223

Composite[b]		
1.	The Bronx, NY	$7,705
2.	White Plains, NY	$7,426
3.	Manhattan, NY	$7,397
4.	Anchorage, AK	$7,318
5.	Huntington, WV	$7,141
6.	Gulfport, MS	$7,135
7.	Abilene, TX	$7,128
8.	East Long Island, NY	$7,116
9.	San Angelo, TX	$7,004
10.	Owensboro, KY	$6,872
11.	Longview, TX	$6,784
12.	Wichita Falls, TX	$6,775
13.	Sioux Falls, SD	$6,775
14.	Bismarck, ND	$6,755
15.	Terre Haute, IN	$6,732
16.	San Mateo County, CA	$6,721
17.	Minot, ND	$6,719
18.	Fort Worth, TX	$6,689
19.	Ridgewood, NJ	$6,682
20.	Beaumont, TX	$6,656

© 2022 Cooper Z et al. *JAMA Network Open.*

(**B**) **Lowest-Spending HRRs by Payor**

Private		
1.	San Bernardino, CA	$2,655
2.	Honolulu, HI	$2,801
3.	Tucson, AZ	$3,032
4.	Rochester, NY	$3,177
5.	Buffalo, NY	$3,227
6.	Royal Oak, MI	$3,261
7.	Takoma Park, MD	$3,297
8.	Fresno, CA	$3,308
9.	San Diego, CA	$3,321
10.	Palm Springs/Rancho Mira, CA	$3,355
11.	Mobile, AL	$3,367
12.	Hattiesburg, MS	$3,374
13.	Petoskey, MI	$3,448
14.	Springfield, MA	$3,457
15.	Baltimore, MD	$3,461
16.	Syracuse, NY	$3,475
17.	Pontiac, MI	$3,484
18.	Boulder, CO	$3,490
19.	Detroit, MI	$3,496
20.	Tuscaloosa, AL	$3,519

Medicare		
1.	Honolulu, HI	$7,654
2.	Grand Junction, CO	$7,920
3.	Asheville, NC	$8,150
4.	Dubuque, IA	$8,176
5.	Missoula, MT	$8,185
6.	Wausau, WI	$8,191
7.	Des Moines, IA	$8,344
8.	Albuquerque, NM	$8,354
9.	Appleton, WI	$8,381
10.	La Crosse, WI	$8,413
11.	Iowa City, IA	$8,511
12.	Madison, WI	$8,522
13.	Boise, ID	$8,522
14.	Neenah, WI	$8,543
15.	Bend, OR	$8,572
16.	Yakima, WA	$8,619
17.	Bloomington, IL	$8,631
18.	Rapid City, SD	$8,632
19.	Medford, OR	$8,658
20.	Green Bay, WI	$8,701

Medicaid		
1.	Springfield, IL	$2,692
2.	Urbana, IL	$2,780
3.	Aurora, IL	$3,023
4.	Bloomington, IL	$3,343
5.	Hinsdale, IL	$3,397
6.	Elgin, IL	$3,476
7.	Rockford, IL	$3,561
8.	Peoria, IL	$3,619
9.	Melrose Park, IL	$3,733
10.	Fort Collins, CO	$3,789
11.	Green Bay, WI	$3,889
12.	Dothan, AL	$3,908
13.	Boulder, CO	$3,977
14.	Chicago, IL	$4,050
15.	Colorado Springs, CO	$4,094
16.	Lawton, OK	$4,107
17.	Montgomery, AL	$4,132
18.	Greeley, CO	$4,213
19.	Evanston, IL	$4,213
20.	Blue Island, IL	$4,215

Composite[b]		
1.	Honolulu, HI	$4,184
2.	Bloomington, IL	$4,392
3.	Boulder, CO	$4,436
4.	San Bernardino, CA	$4,685
5.	Hinsdale, IL	$4,757
6.	Tucson, AZ	$4,765
7.	Olympia, WA	$4,768
8.	Colorado Springs, CO	$4,777
9.	Pueblo, CO	$4,799
10.	Palm Springs/Rancho Mira, CA	$4,813
11.	San Diego, CA	$4,841
12.	Fresno, CA	$4,852
13.	Springfield, IL	$4,864
14.	Seattle, WA	$4,869
15.	Reno, NV	$4,902
16.	Evanston, IL	$4,914
17.	Petoskey, MI	$4,919
18.	Peoria, IL	$4,941
19.	Montgomery, AL	$4,965
20.	Mobile, AL	$4,976

[a]All spending measures are age- and sex-adjusted using indirect standardization and presented in 2017 USD.
[b]Composite spending is an average of private, Medicare, and Medicaid spending, weighted by the state-level share of the population that is insured by the payor.

© 2022 Cooper Z et al. *JAMA Network Open.*

eTable 3. Correlation of Inclusive and Exclusive Medicaid Spending Per Beneficiary and Spending per Beneficiary Across Payors with an Inclusive Estimate of Medicaid Spending [a]

**(A) Correlation of Medicaid Spending Per Beneficiary (Age- and Sex-Adjusted)**			**(B) Correlation of Spending Per Beneficiary (Age- and Sex-Adjusted)**			
				Private	Medicare	Medicaid (Incl) [b]
	Medicaid (Incl)[b]		Private	1.000		
Medicaid (Excl)	0.931***		Medicare	0.020		
			Medicaid (Incl) [b]	0.236***	0.198***	

[a]HRR-level spending measures are age- and sex-adjusted using indirect standardization, and inflation-adjusted to 2017 USD.
[b]The inclusive estimate of Medicaid spending reintroduces individuals without full benefits and without full eligibility group information.

© 2022 Cooper Z et al. *JAMA Network Open.*

eFigure 1. Scatter Plots of Correlations of HRR-Level Spending Per Beneficiary Across Payors

(A)

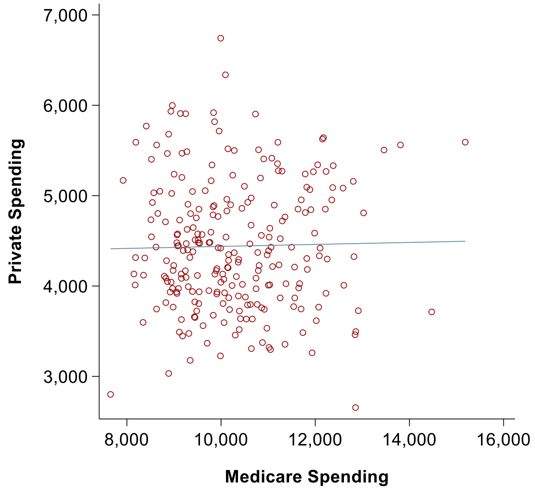

Correlation: 0.020

(B)

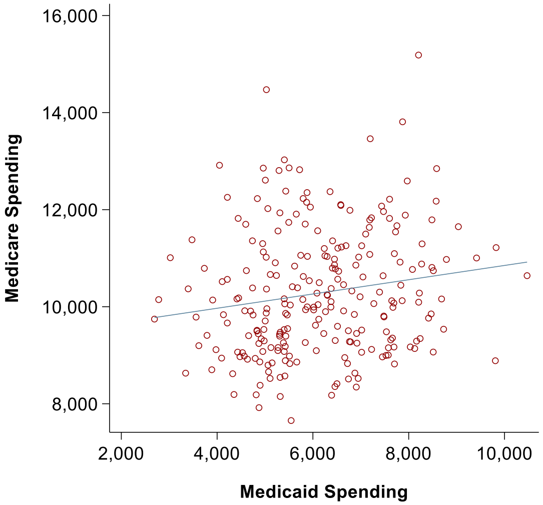

Correlation: 0.162**

(C)

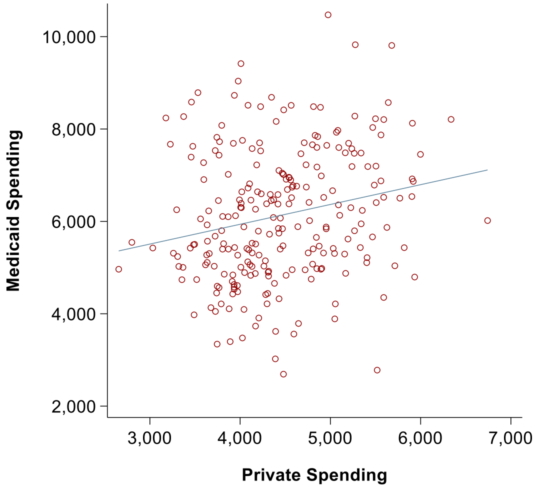

Correlation: 0.213***

Notes: HRR-level spending measures are age- and sex-adjusted using indirect standardization, and inflation-adjusted to 2017 USD.

© 2022 Cooper Z et al. *JAMA Network Open.*

eFigure 2. Bivariate Correlates of Spending and Inpatient Bed-Days Per Beneficiary Across Payors

(A) Spending Per Beneficiary

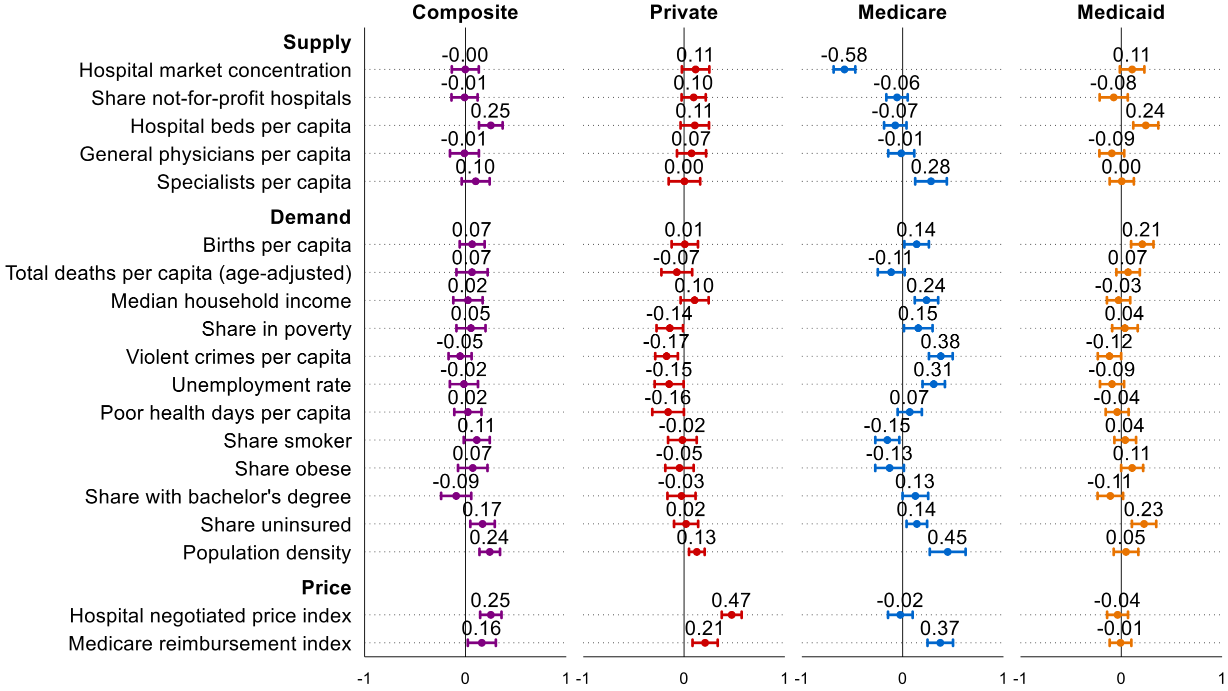

(B) Inpatient Bed-Days Per Beneficiary

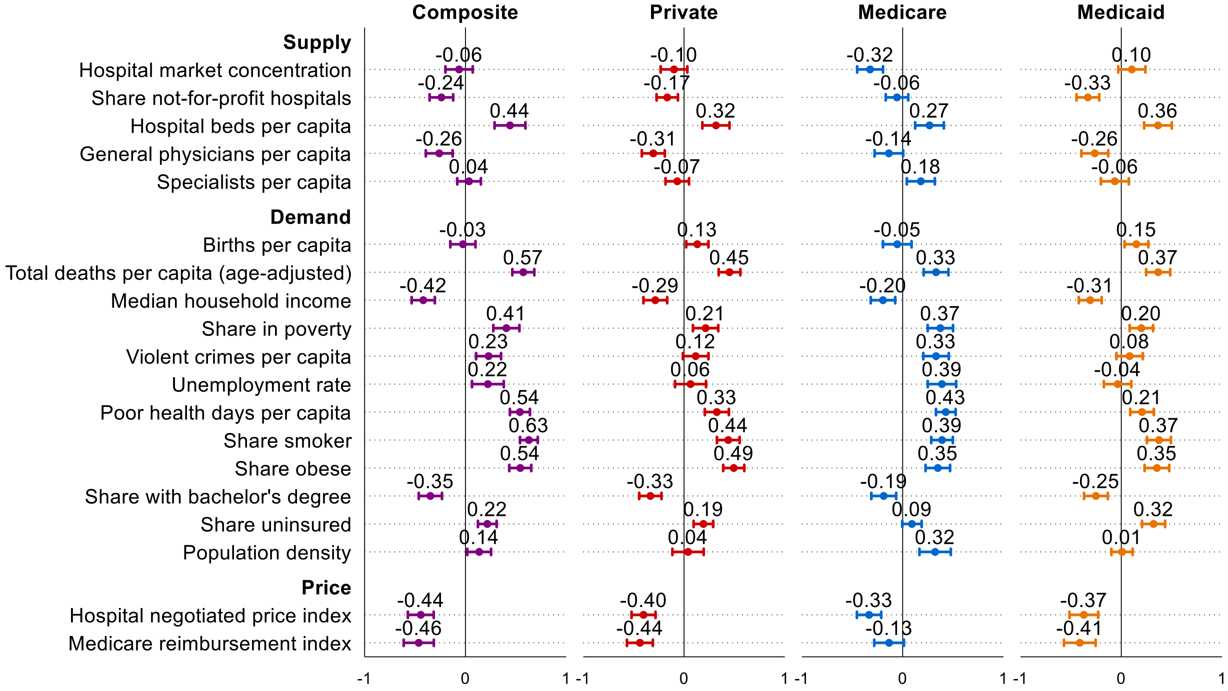

Notes: The bivariate correlates include 95% confidence intervals. HRR-level spending and inpatient bed-days per beneficiary are age- and sex-adjusted using indirect standardization. Composite spending and bed-days are an average of private, Medicare, and Medicaid spending and bed-days, respectively, weighted by the state-level share of the population that is insured by the payor.

© 2022 Cooper Z et al. *JAMA Network Open.*

eFigure 3. Bivariate Correlates of Spending and Inpatient Bed-Days Per Beneficiary Across Payors with Characteristics Selected by a LASSO Regression

(A) Spending Per Beneficiary

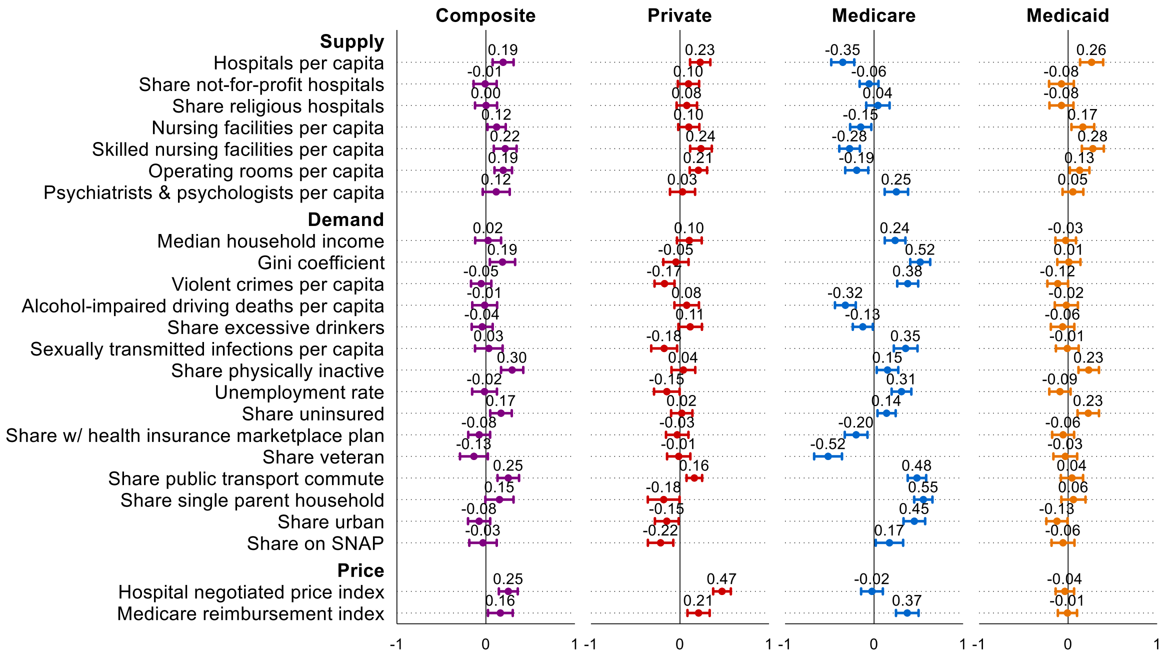

(B) Inpatient Bed-Days Per Beneficiary

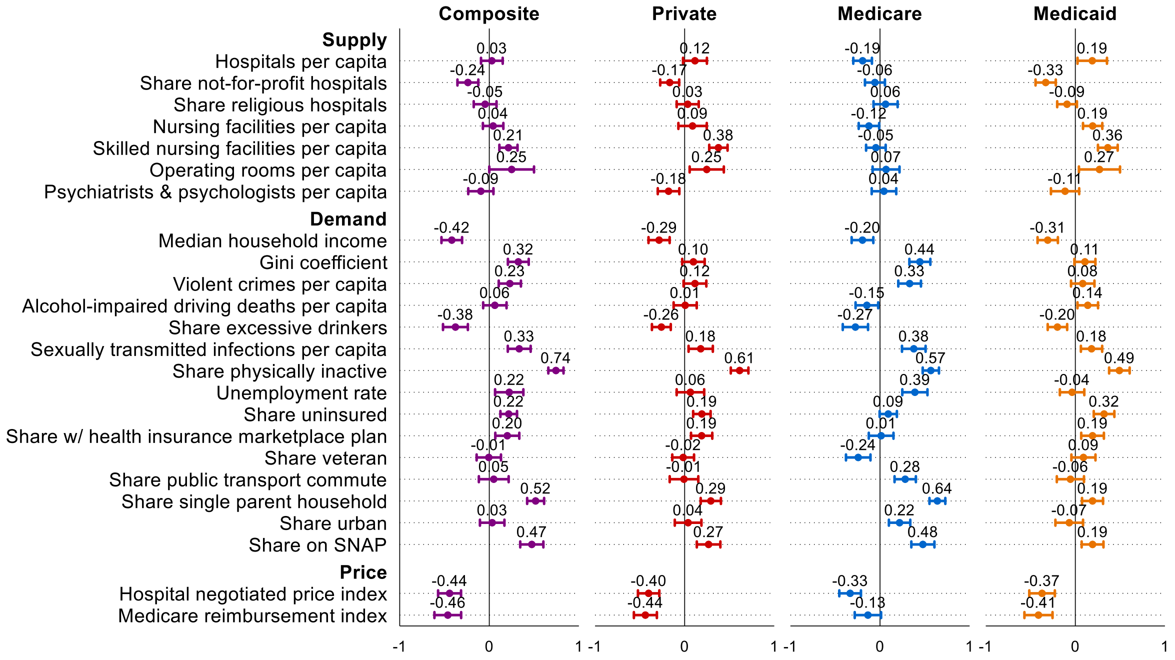

Notes: The characteristics were selected by a LASSO regression on composite spending, with the penalizing parameter applied to the coefficient of the explanatory variables included in the regression chosen by 10-fold cross validation to minimize the mean squared error. The bivariate correlates include 95% confidence intervals. HRR-level spending and inpatient bed-days per beneficiary are age- and sex-adjusted using indirect standardization. Composite spending and bed-days are an average of private, Medicare, and Medicaid spending and bed-days, respectively, weighted by the state-level share of the population that is insured by the payor.

© 2022 Cooper Z et al. *JAMA Network Open.*

eFigure 4. Bivariate Correlates of Spending and Inpatient Bed-Days Per Beneficiary Across Payors Using a Bonferroni Correction

(A) Spending Per Beneficiary

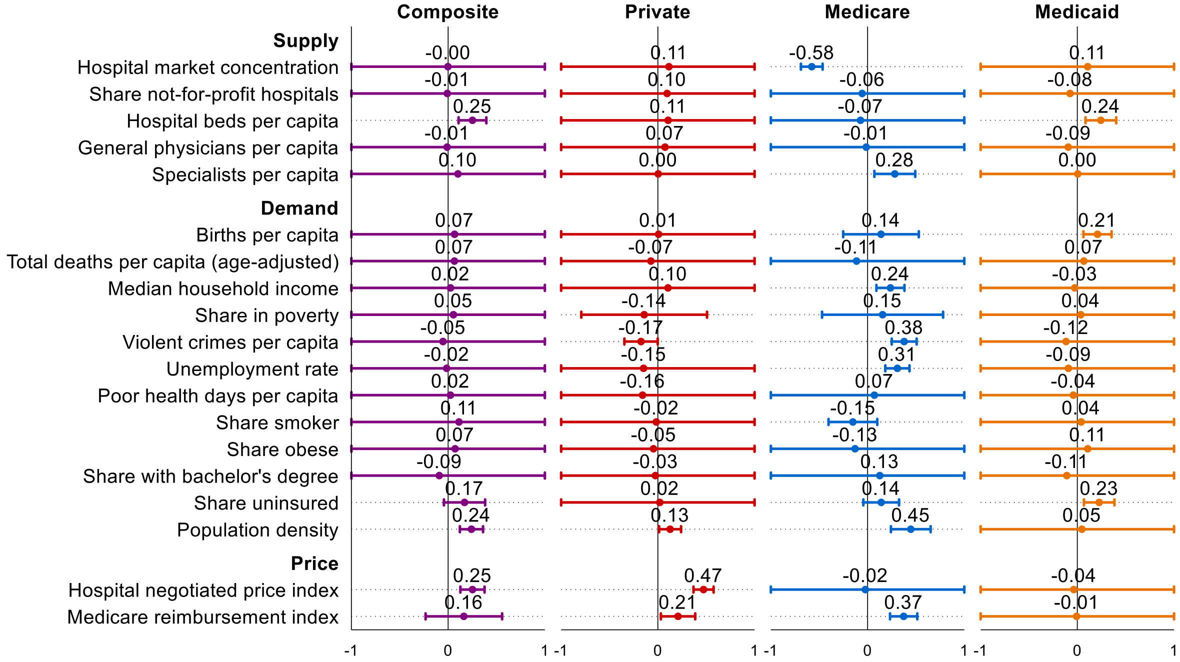

(B) Inpatient Bed-Days Per Beneficiary

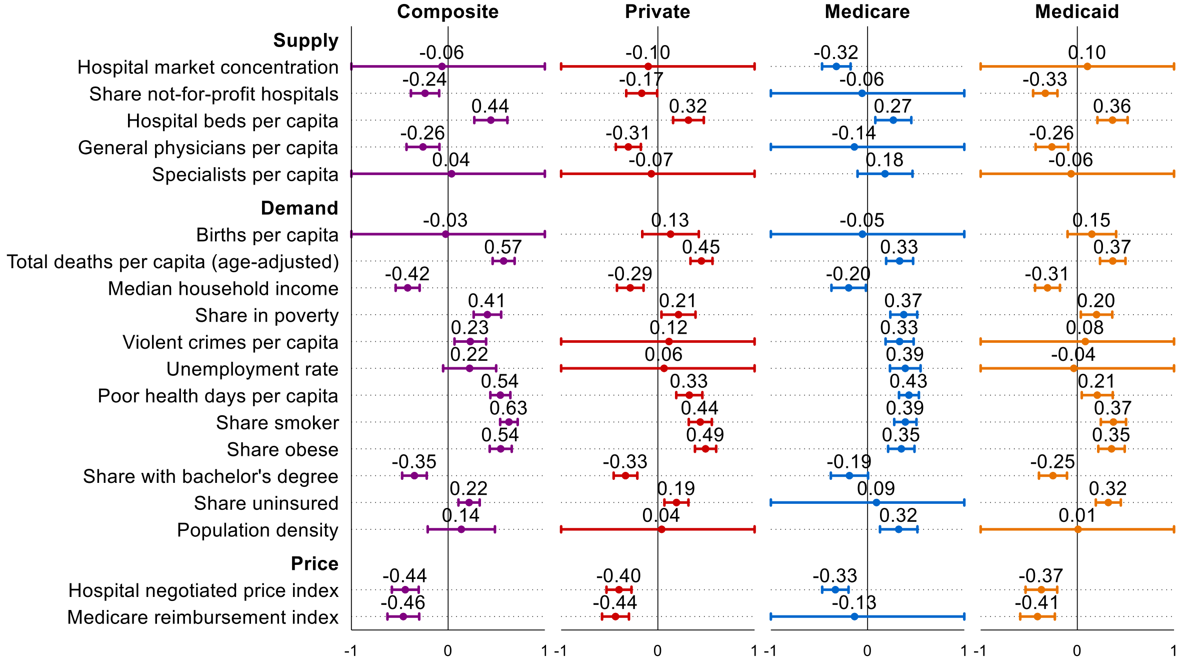

Notes: The bivariate correlates include 95% confidence intervals under a Bonferroni correction to the p-values. HRR-level spending and inpatient bed-days per beneficiary are age- and sex-adjusted using indirect standardization. Composite spending and bed-days are an average of private, Medicare, and Medicaid spending and bed-days, respectively, weighted by the state-level share of the population that is insured by the payor.

© 2022 Cooper Z et al. *JAMA Network Open.*
